# Selective use of reinforced tissue matrix in complex abdominal wall reconstruction: a multicenter consensus analysis

**DOI:** 10.3389/jaws.2026.17323

**Published:** 2026-09-22

**Authors:** Sebastian Schaaf, Thomas Mones, Peter Mai, Niels-Torsten Hoedt, Markus Götz, Arnulf Willms, Guido Woeste

**Affiliations:** 1 Bundeswehrzentralkrankenhaus Koblenz, Department of General, Visceral and Thoracic Surgery, Koblenz, Germany; 2 Städtisches Krankenhaus Maria-Hilf Brilon gGmbH, Brilon, Germany; 3 Barmherzige Brüder Krankenhaus München, Munich, Germany; 4 Evangelisches Diakonissenhaus, Leipzig, Germany; 5 Universitätsklinikum Regensburg, Regensburg, Germany; 6 Agaplesion Elisabethenstift gGmbH, Darmstadt, Germany

**Keywords:** bridging, complex abdominal wall reconstruction (CAWR), contamination, incisional hernia repair, infection

## Abstract

**Background:**

Optimal mesh selection in complex abdominal wall reconstruction remains controversial, particularly in contaminated fields or in patients with extensive tissue loss. Reinforced tissue matrix (RTM) meshes combine biologic material with permanent synthetic reinforcement and may represent a potential option in selected cases. This study aimed to analyze clinical use patterns of RTM meshes and identify potential indication niches through structured expert consensus.

**Methods:**

A multicenter expert panel collected cases of abdominal wall reconstruction using RTM mesh (OviTex). Patient characteristics, hernia features, operative strategies, and postoperative outcomes were recorded in a standardized database. Cases were evaluated according to established complexity criteria. Seven abdominal wall surgeons independently rated the appropriateness of RTM use. Consensus was defined as ≥70% agreement, and interrater reliability was assessed using Fleiss’ kappa.

**Results:**

Seventy-four patients were included (mean age 63.9 ± 12.0 years; median BMI 30.2 kg/m^2^). Most patients presented with incisional hernias and large fascial defects. Contaminated or potentially contaminated operative fields were present in approximately one-third of cases. Surgical site infection occurred in 20.3%, mesh infection in 7.8%, and hernia recurrence in 12.3%. Expert consensus supporting RTM implantation was reached in 50% of cases, particularly in scenarios combining contamination with large fascial defects. Interrater agreement was fair (κ = 0.34).

**Conclusion:**

Routine use of RTM meshes in abdominal wall reconstruction is not supported. However, RTM may represent a potential option in selected high-complexity scenarios involving contamination and large fascial defects where permanent synthetic meshes may be unsuitable. These findings are exploratory and hypothesis-generating and may support future Delphi-based indication consensus development.

## Introduction

Complex abdominal wall reconstruction (CAWR) remains a major challenge in abdominal wall surgery, particularly in patients with large fascial defects, contamination, prior mesh infection, loss of domain, or multiple previous repairs [1–3]. These complex clinical scenarios are associated with increased risks of surgical site complications, recurrence, and impaired wound healing. Current classification systems therefore define complex abdominal wall hernias based on defect characteristics, contamination, patient-related risk factors, and surgical history, providing a framework for reconstructive planning and outcome assessment [1].

Permanent synthetic meshes remain the standard material for ventral and incisional hernia repair because of their favorable long-term durability and low recurrence rates [2]. However, their use in contaminated or potentially contaminated operative fields remains controversial because of the risk of chronic infection, mesh-related complications, and explantation [4, 5]. Current European Hernia Society guidelines therefore emphasize careful patient selection, infection control, and optimization of the surgical field in complex abdominal wall reconstruction [2]. In addition, bridging repairs without primary fascial closure are generally debated because of their high recurrence rates [6].

Biologic meshes were introduced to improve tissue integration and potentially reduce infection-related complications [7]. However, accumulating evidence has questioned their long-term durability and clinical benefit, particularly in bridging situations and large fascial defects. Randomized and comparative studies have demonstrated higher recurrence rates and substantial costs associated with biologic meshes compared with synthetic materials, even in contaminated ventral hernia repair [8, 9]. Fully or slowly absorbable biosynthetic meshes represent another alternative, particularly in contaminated or high-risk settings [10–12]. However, evidence for their use in highly complex abdominal wall reconstruction, particularly in scenarios involving major tissue loss or the potential need for bridging, remains limited.

To address these limitations, reinforced tissue matrices (RTMs) have been developed as hybrid implants combining biologic extracellular matrix scaffolds with permanent synthetic reinforcement [13]. OviTex® (Tela Bio Inc., Malvern, PA, USA) represents one such RTM designed to combine biologic integration with improved mechanical stability. Early clinical studies suggest potential benefits in selected high-risk patients and contaminated operative fields, although evidence remains limited and clear indication criteria are lacking [14–16].

The present study therefore aimed to characterize the clinical scenarios in which RTM meshes have been used in complex abdominal wall reconstruction and to explore whether these indications are considered appropriate by a structured expert panel. The objective was not to assess superiority or non-inferiority of RTM compared with synthetic, biologic, or biosynthetic meshes, but to identify clinical scenarios in which experts perceive a potential indication for RTM use. The study should therefore be interpreted as exploratory and hypothesis-generating, with the goal of informing future comparative studies and evidence-based indication development.

## Materials and methods

### Study design and patient cohort

This study was designed as a retrospective multicenter case series combined with a structured expert consensus analysis. Participating abdominal wall surgery centers contributed cases of complex abdominal wall reconstruction in which a reinforced tissue matrix (RTM) mesh (OviTex®, Tela Bio Inc., Malvern, PA, USA) had been implanted.

Clinical data were entered into a standardized database and included patient demographics, comorbidities, abdominal wall defect characteristics, operative details, perioperative findings, and postoperative outcomes. Recorded variables included age, sex, body mass index (BMI), ASA classification, comorbidities associated with surgical site occurrence (SSO) or recurrence risk, defect type and size, contamination status, operative technique, mesh type and size, postoperative complications, recurrence, mortality, and follow-up duration.

Contamination status was classified according to the Centers for Disease Control and Prevention (CDC) surgical wound classification. Class I was considered clean, class II clean-contaminated, class III contaminated, and class IV dirty/infected. For descriptive analyses, the term ‘contaminated or potentially contaminated’ encompassed CDC classes II–IV.

Mesh infection/colonization was defined as a clinically suspected mesh-associated infection with local signs of infection and/or microbiological evidence of microbial colonization involving the mesh or the mesh-containing wound.

### Definition of complex abdominal wall reconstruction

Cases were categorized according to established abdominal wall complexity criteria described in the literature [1, 3]. Complexity determinants were grouped into four domains: defect size and location, contamination or soft tissue condition, patient-related risk factors, and clinical scenario or surgical history. Individual cases were assessed based on documented operative and clinical findings.

Large fascial defects and true loss of abdominal wall substance were considered distinct morphological complexity features. However, for the indication analysis, these scenarios were grouped when they resulted in a residual abdominal wall defect that could not be closed primarily despite appropriate reconstructive measures and therefore required mesh-based reconstruction or bridging. Thus, the combined indication category reflects the reconstructive problem rather than implying morphological equivalence between a large fascial defect and true tissue loss.

Open abdomen-related reconstruction comprised two distinct clinical scenarios [1]: definitive reconstruction during an ongoing open abdomen treatment episode when primary fascial closure could not be achieved, resulting in a residual ventral abdominal wall defect requiring RTM bridging; and [2] secondary abdominal wall reconstruction of a planned ventral hernia resulting from previous open abdomen treatment. Accordingly, a history of open abdomen treatment was not considered an indication for RTM use *per se*, but represented a complexity feature when associated with a residual abdominal wall defect requiring subsequent reconstruction.

### Expert consensus assessment

Seven experienced abdominal wall surgeons independently reviewed all cases and evaluated the appropriateness of RTM implantation. All panel members had contributed cases to the study cohort and had clinical experience with RTM implantation. Each expert evaluated the complete case series, including cases contributed by their own institution. Accordingly, the assessment represents a structured evaluation among surgeons experienced with RTM rather than an independent external validation of its indications.

For the consensus assessment, each case was presented to the expert panel using a standardized case summary including relevant patient characteristics, comorbidities, abdominal wall defect characteristics, contamination status, previous abdominal wall surgery and mesh-related complications, and the operative/reconstructive setting. The experts were asked to assess retrospectively whether, based on the clinical information available for each case, the use of an RTM represented a reasonable reconstructive option. Importantly, the assessment did not address whether RTM was superior to alternative mesh materials, but whether its use was considered justifiable within the respective clinical scenario.

Ratings were recorded as binary variables (0 = no indication, 1 = indication for RTM use). Non-binary or incomplete responses were excluded from analysis. Consensus was predefined as agreement among at least 70% of raters (five of seven) either supporting or opposing RTM implantation. In addition, cases were stratified according to the primary documented indication for RTM use to assess indication-specific consensus patterns.

### Outcome measures

The primary outcome was expert agreement regarding the appropriateness of RTM use within the respective clinical scenario. Additional descriptive outcomes included surgical site infection (SSI), mesh infection, hernia recurrence, reoperation, mortality, operative strategies, contamination status, and complexity criteria associated with RTM implantation.

### Follow-up and recurrence assessment

Follow-up was based on routine clinical care and was therefore not standardized across participating centers. Patients attending clinical follow-up underwent physical examination and generally abdominal wall ultrasonography. In some patients, follow-up information was obtained by telephone; patients reporting symptoms suggestive of recurrence were invited for further clinical assessment. Hernia recurrence was defined as a clinically or radiologically detected recurrent abdominal wall defect at the site of the index reconstruction. Systematic imaging at predefined intervals was not mandated.

### Statistical analysis

Statistical analyses were performed using Microsoft Excel, SPSS version 26 (IBM Corp., Armonk, NY, USA), and R (R Foundation for Statistical Computing, Vienna, Austria). Continuous variables were assessed for normality using the Shapiro–Wilk test and are presented as mean ± standard deviation (SD) or median with range, as appropriate. Categorical variables are reported as absolute numbers and percentages.

Interrater agreement among expert raters was assessed using Fleiss’ kappa statistic for multiple raters. Kappa values were interpreted according to the Landis and Koch classification.

### Ethical approval

The study involving human participants was reviewed and approved by the Ethics Committee of the University of Regensburg (reference no. 26-4640-104). The study was performed in accordance with the ethical standards of the Declaration of Helsinki and its later amendments. Due to the retrospective nature of the study and the use of anonymized clinical data, the requirement for written informed consent was waived by the Ethics Committee.

## Results

### Patient characteristics

A total of 74 patients were included (Table 1). Mean age was 63.9 ± 12.0 years, and median body mass index (BMI) was 30.2 kg/m^2^ (range 17.0–54.0). The cohort consisted of 41 women (55.4%) and 33 men (44.6%). Overall, 76.7% of patients presented with at least one risk factor associated with surgical site occurrence or recurrence. The most common comorbidities were obesity (30.1%), diabetes mellitus (28.8%), nicotine abuse (27.4%), and chronic obstructive pulmonary disease (19.2%). Most patients were classified as ASA II (52.7%) or ASA III (40.5%).

**TABLE 1:** Baseline characteristics and operative setting of the study cohort (n = 74).

Category	Variable	Value
Demographics	Age, years	63.9 ± 12.0
	Female sex	41 (55.4%)
	Male sex	33 (44.6%)
	BMI, kg/m^2^	30.2 (17.0–54.0)
ASA classification	ASA I	1 (1.4%)
	ASA II	39 (52.7%)
	ASA III	30 (40.5%)
	ASA IV	4 (5.4%)
Risk profile	Patients with ≥1 risk factor	56 (76.7%)
Comorbidities	Obesity	22 (30.1%)
	Diabetes mellitus	21 (28.8%)
	Nicotine abuse	20 (27.4%)
	COPD	14 (19.2%)
	Asthma	4 (5.5%)
	Liver cirrhosis	2 (2.7%)
	Other	≤1 (≤1.4%)
Primary indication for surgery	Hernia repair	61 (82.4%)
	Laparostoma	9 (12.2%)
	Other	4 (5.4%)
Wound classification (CDC)	Clean (I)	37 (50.0%)
	Clean-contaminated (II)	23 (31.1%)
	Contaminated (III)	11 (14.9%)
	Dirty/infected (IV)	3 (4.1%)

### Hernia characteristics and operative indications

The primary indication for surgery was hernia repair in 82.4% of cases, followed by open abdomen closure in 12.2%. According to CDC wound classification, 50.0% of procedures were classified as clean (class I), 31.1% as clean-contaminated (class II), 14.9% as contaminated (class III), and 4.1% as dirty/infected (class IV).

Based on the European Hernia Society classification, midline hernias were present in 86.5% and lateral hernias in 20.3% of cases. Median hernia width was 11 cm (range 0–30 cm), and median defect area was 129 cm^2^ (range 0–900 cm^2^). Among patients with available width classification, 56.9% were classified as W3 defects.

Recurrent hernias were present in 20.3%, frequently after previous mesh repair. Additional complex scenarios included loss of domain, prior open abdomen treatment, mesh infection, enterocutaneous fistula, and incarcerated hernia with bowel obstruction. Overall, most patients fulfilled multiple complexity criteria across several domains of the abdominal wall complexity framework, particularly defect size, contamination-related conditions, and previous surgical repair history (Table 2).

**TABLE 2:** Distribution of complexity criteria according to the abdominal wall complexity framework according to Capoccia Giovannini et al. https://doi.org/10.1093/bjs/znad346.

Domain	Complexity criterion	n (%)
Defect size/location	Large defect (width)	24 (32.4%)
	Incisional flank/lateral hernia	10 (13.5%)
	Loss of domain	3 (4.1%)
	Loss of muscles/missing muscular support	3 (4.1%)
	Parastomal/stoma-related	2 (2.7%)
Contamination/soft tissue condition	Abdominal wall or mesh infection	9 (12.2%)
	Open abdomen/laparostoma	7 (9.5%)
	Fistula	6 (8.1%)
	Skin defect/chronic wound infection	4 (5.4%)
Patient-related factors	BMI/obesity	9 (12.2%)
	Cirrhosis with ascites	2 (2.7%)
	Severe comorbidity (ASA)	1 (1.4%)
Clinical scenario/surgical history	Multiple previous repairs	14 (18.9%)
	Recurrent hernia	13 (17.6%)
	Previous open abdomen	2 (2.7%)
	Stoma present	2 (2.7%)

### Indications for reinforced tissue matrix implantation

The most commonly used implant was OviTex 2S (60.8%), followed by OviTex LPR (20.3%). Mesh sizes ranged from 10 × 12 cm to 45 × 25 cm.

The documented indications for RTM implantation are summarized in Table 3. The most frequent indication was large fascial defects or tissue loss (40.5%), followed by contaminated or potentially contaminated operative fields (31.1%). Additional indications included high-risk patient profiles (16.2%), previous mesh implantation or recurrent hernia (14.9%), bridging situations or loss of domain (9.5%), and open abdomen or laparostoma reconstruction (8.1%).

**TABLE 3:** Indications for reinforced tissue matrix mesh implantation were categorized based on the documented surgical rationale. Multiple indication categories could apply to a single patient; therefore, percentages may exceed 100%.

Indication for reinforced tissue matrix mesh	n (%)
Large fascial defect/tissue loss	30 (40.5%)
Contaminated or potentially contaminated surgical field	23 (31.1%)
High-risk patient (e.g., obesity, COPD, diabetes, nicotine abuse)	12 (16.2%)
Previous mesh implantation or recurrence	11 (14.9%)
Bridging situation/loss of domain	7 (9.5%)
Open abdomen/laparostoma reconstruction	6 (8.1%)

### Operative procedures

Intraperitoneal onlay mesh (IPOM) repair was the most frequently performed technique (46.6%), followed by retromuscular reconstruction (20.5%). Bridging repair was required in 12.3% of patients. Advanced reconstructive strategies including transversus abdominis release (TAR), component separation, fascial traction, botulinum toxin application, and staged reconstruction using negative pressure wound therapy were performed in 16.4% of cases.

### Postoperative outcomes

Table 4 summarized the outcome findings. Median hospital stay was 8 days (range 2–83 days). In-hospital mortality was 4.1%. Surgical site infection occurred in 20.3% of patients with available follow-up.

**TABLE 4:** Values are presented as median (range) or number (percentage). Surgical site infections (SSI), mesh infections and recurrence rates are reported for patients with available follow-up data (*n = 65*).

Outcome	Value
Length of hospital stay, days	8 (2–83)
In-hospital mortality	3 (4.1%)
Surgical site infection (SSI)*	13/65 (20.0%)
SSI ≤30 days	5 (7.8%)
SSI >30 days	8 (12.5%)
Mesh infection	5 (7.8%)
Hernia recurrence	8 (12.3%)
Reoperation due to recurrence	3 (4.6%)
Conservative management of recurrence	5 (7.8%)
No recurrence	57 (87.7%)

Mesh infection/colonization was observed in 5/64 patients (7.8%). All infections were managed without operative revision or mesh explantation using local wound management, including partial wound opening and drainage where required. In one patient with a nearby urostomy, *Candida* colonization/infection involving the mesh was microbiologically confirmed and successfully treated with an eight-week course of fluconazole without subsequent mesh explantation.

Major complications (Clavien–Dindo ≥ IIIb) occurred in 38.3% of patients with available complication data.

Complete follow-up data were available for 65 of 74 patients (87.8%), with a median follow-up duration of 12 months (range 1–36 months). Hernia recurrence occurred in 12.3% of patients, including 3.1% requiring reoperation.

### Expert consensus assessment

A total of 511 valid ratings from seven expert surgeons were analyzed (Table 5). Seven individual ratings were unavailable or incomplete and were excluded according to the predefined analysis rules. Overall, 68.9% of ratings supported RTM implantation. Using the predefined threshold of ≥70% agreement, consensus supporting RTM use was reached in 37 cases (50.0%), whereas 34 cases (45.9%) showed no clear consensus. Consensus against RTM implantation was reached in 3 cases (4.1%).

**TABLE 5:** Expert consensus stratified by indication. Cases were grouped according to the documented clinical indication for OviTex implantation. Consensus was defined as ≥70% agreement among experts and categorized as consensus for OviTex, no consensus, or consensus against OviTex. The highest agreement was observed in cases involving infection or contamination and infection combined with augmentation, whereas substantially lower agreement was found for augmentation/bridging without infection and other indications. Overall interrater agreement among the experts was κ = 0.34, indicating fair agreement according to the Landis and Koch classification.

Indications	Consensus overall	Consensus pro	Consensus against
Augmentation/bridging	17	16	1
Infection/contamination	11	3	0
Infection + augmentation	8	1	0
Others	1	14	2

Agreement was highest in cases involving infection or contamination, particularly when combined with large fascial defects. In contrast, augmentation or bridging without infection demonstrated substantially lower consensus rates. Overall interrater agreement was fair (Fleiss’ κ = 0.34). The fair interrater agreement should be interpreted in the context of a heterogeneous real-world cohort and likely reflects indication-dependent decision making rather than a uniformly applied RTM indication algorithm.

## Discussion

The present study provides insight into current expert perception and clinical use patterns of reinforced tissue matrix (RTM) meshes in complex abdominal wall reconstruction. The highest level of agreement supporting RTM implantation was observed in cases combining contamination or infection with large fascial defects. In contrast, substantially lower consensus was observed in augmentation or bridging situations without contamination. These findings suggest that RTM meshes are currently perceived not as routine implants, but rather as selective options for highly complex reconstructive scenarios.

The relatively high proportion of intraperitoneal mesh placement reflects the complexity and reconstructive constraints of the selected cohort. Intraperitoneal placement was primarily used in cases in which retromuscular reconstruction was considered technically unsuitable or not feasible, including destruction or insufficient integrity of the posterior abdominal wall layers, extensive previous surgery, and complex open abdomen or contaminated settings.

Bridging repair was restricted to cases in which primary fascial closure could not be achieved or was considered unsuitable despite available reconstructive measures. Depending on the individual clinical situation, attempts at fascial medialization included component separation, fascial traction, botulinum toxin application, and/or staged reconstruction. Bridging was therefore generally used as a salvage reconstructive strategy rather than as the preferred primary approach.

Importantly, the present findings support the concept that complex abdominal wall reconstruction requires individualized and tailored reconstructive strategies rather than a uniform mesh-based approach [1, 2, 17]. Defect morphology, contamination status, soft tissue quality, prior surgical history, and patient-related risk factors must all be integrated into surgical decision-making [1, 3, 18]. In many patients, successful reconstruction depends less on the choice of a specific mesh and more on the ability to combine appropriate reconstructive techniques with adequate infection control and restoration of fascial continuity.

Permanent synthetic meshes remain the standard material for ventral and incisional hernia repair because of their favorable long-term durability and low recurrence rates [2]. However, their use in contaminated or potentially contaminated operative fields remains controversial because of the risk of chronic infection, mesh-related complications, and explantation [2, 4, 5, 8]. This concern was also reflected in the present expert consensus analysis, in which the strongest agreement supporting RTM implantation was observed in contaminated settings and infection-associated abdominal wall defects.

Several studies have questioned the long-term benefit of biologic meshes in abdominal wall reconstruction. Rosen et al. demonstrated significantly higher recurrence rates for biologic meshes compared with synthetic meshes in contaminated ventral hernia repair despite substantially higher treatment costs [8]. Similarly, Köckerling et al. emphasized the limited evidence supporting biologic meshes, particularly in bridging situations and large fascial defects [9]. These findings reinforce current recommendations favoring macroporous synthetic meshes in most standard reconstructive scenarios because of their superior durability and cost-effectiveness [2, 5].

Alternative strategies have therefore been explored for patients with increased infection risk. Biosynthetic and absorbable meshes may represent one such option [17, 18]. Meta-analytic data suggest potentially favorable recurrence and surgical site infection rates in contaminated ventral hernia repair, although evidence remains limited by the absence of robust comparative trials [4]. Within this context, RTM meshes aim to combine biologic integration with improved mechanical stability through permanent synthetic reinforcement [6, 14–16].

Importantly, the present study should not be interpreted as supporting routine bridging reconstruction using RTM meshes. The indication patterns summarized in Table 3 may provide a preliminary phenotype-oriented framework for future investigation of RTM use in complex abdominal wall reconstruction. Current abdominal wall reconstruction principles emphasize that primary fascial closure should be achieved whenever possible [2, 5]. Advanced reconstructive strategies including botulinum toxin application, intraoperative fascial traction, component separation techniques, and transversus abdominis release should therefore be considered before accepting bridging repair, particularly in patients with large defects or loss of domain [2, 6]. All participating centers routinely employ the full spectrum of adjunctive techniques for fascial medialization, including component separation, fascial traction, botulinum toxin application, and staged reconstruction, as appropriate to the individual clinical scenario. Bridging repair was restricted to cases in which primary fascial closure could not be achieved or was considered unsuitable despite available reconstructive measures. The choice and combination of adjunctive techniques were individualized and were not standardized by a study-specific protocol. Bridging therefore represented a salvage reconstructive strategy rather than a preferred primary approach.

Within this reconstructive framework, RTM meshes may represent a potential option in situations where conventional permanent synthetic meshes are considered unfavorable because of contamination, prior mesh infection, compromised soft tissue conditions, or anticipated difficulties with mesh salvage in the event of postoperative infection [6]. The hybrid design aims to combine biologic integration with greater structural durability than purely biologic implants [13]. Early clinical studies have reported acceptable recurrence rates and favorable mesh salvage potential in selected high-risk patients undergoing abdominal wall reconstruction with RTM meshes [14, 16]. Although no conclusions can be drawn from this retrospective cohort, the absence of mesh explantation despite documented mesh infections may warrant further investigation of mesh salvage as a clinically relevant outcome. Nevertheless, evidence remains limited, and clear evidence-based indication criteria are currently lacking.

Current evidence does not support routine use of biologic or hybrid meshes in standard ventral hernia repair. Consequently, RTM implantation should presently be regarded as a selective reconstructive strategy for specific high-complexity situations rather than as a general alternative to synthetic mesh repair. The consensus patterns observed in the present study support this interpretation. The highest agreement was observed in cases involving contamination, infection, and major tissue loss, whereas substantially lower consensus was seen in standard augmentation scenarios without contamination or major soft tissue compromise. Thus, consensus was not uniformly distributed across all cases but appeared to cluster in specific high-complexity phenotypes.

This study should be interpreted as exploratory and hypothesis-generating rather than as evidence supporting routine RTM use. The aim of the present analysis was not to advocate widespread application of RTM meshes, but rather to identify potential indication niches in which conventional reconstructive strategies may be suboptimal. These findings may therefore serve as a starting point for a structured international Delphi consensus process aimed at defining evidence-informed indications for RTM implantation in complex abdominal wall reconstruction.

Importantly, the clinical outcomes observed in this cohort cannot establish whether RTM performs better, similarly, or worse than permanent synthetic, biologic, or biosynthetic meshes. Such comparisons would require adequately controlled comparative studies with standardized indications and follow-up. The outcome data presented here primarily characterize the clinical context in which RTM was used and provide descriptive safety signals rather than comparative evidence of effectiveness.

Future comparative studies should therefore specifically evaluate RTM against contemporary permanent synthetic and long-term absorbable biosynthetic meshes within clearly defined high-complexity phenotypes, particularly contaminated reconstruction and large defects requiring advanced abdominal wall reconstruction.

### Limitations

Several limitations of this study should be acknowledged. First, the analysis is based on a retrospective multicenter case collection and therefore reflects selected clinical scenarios rather than a consecutive patient cohort. Second, surgical techniques and reconstructive strategies were heterogeneous, reflecting real-world decision-making in complex abdominal wall reconstruction but limiting direct comparison between approaches. Third, follow-up duration was variable and relatively limited in some patients, which may underestimate long-term recurrence rates. In addition, the absence of a comparative control group precludes conclusions regarding superiority or equivalence compared with alternative reconstructive materials. Finally, the expert consensus analysis reflects the judgment of a limited number of abdominal wall surgeons and is inherently susceptible to expert-selection and preference bias. Individual experience with RTM, biologic, biosynthetic, and permanent synthetic meshes, as well as different institutional approaches to mesh use in contaminated fields, may have influenced the ratings. Moreover, all experts had contributed cases to the study and also assessed cases originating from their own institutions, introducing a potential confirmation bias. The consensus assessment should therefore not be interpreted as an objective validation of RTM indications, but as an exploratory assessment of current expert perception.

Furthermore, follow-up assessment was not standardized and did not include systematic imaging in all patients. Therefore, asymptomatic or occult recurrences may have remained undetected, and the reported recurrence rate should primarily be interpreted as reflecting clinically detected recurrence. The relatively short median follow-up of 12 months also limits assessment of late recurrence and late mesh-related infection.

## Conclusion

The present study does not provide evidence supporting the routine use of reinforced tissue matrix meshes in abdominal wall reconstruction. Instead, the findings suggest that RTM meshes may occupy specific indication niches, particularly in complex situations combining contamination, previous infection, and large fascial defects where conventional synthetic meshes may be suboptimal. RTM implantation should therefore be considered within a tailored reconstructive strategy aimed at achieving durable fascial closure while minimizing infection-related complications. These findings are exploratory and hypothesis-generating and may serve as a foundation for future international Delphi consensus development regarding evidence-informed indications for RTM use in complex abdominal wall reconstruction.

## Data Availability

The raw data supporting the conclusions of this article will be made available by the authors, without undue reservation.

## References

[1] Capoccia Giovannini S Podda M Ribas S Montori G Botteri E Agresta F What defines an incisional hernia as 'complex': results from a Delphi consensus endorsed by the european hernia society (EHS). Br J Surg (2024) 111(1):znad346. 10.1093/bjs/znad346 37897716

[2] Sanders DL Pawlak MM Simons MP Aufenacker T Balla A Berger C Midline incisional hernia guidelines: the european hernia society. Br J Surg (2023) 110(12):1732–68. 10.1093/bjs/znad284 37727928 PMC10638550

[3] Slater NJ Montgomery A Berrevoet F Carbonell AM Chang A Franklin M Criteria for definition of a complex abdominal wall hernia. Hernia (2014) 18(1):7–17. 10.1007/s10029-013-1168-6 24150721

[4] Quiroga-Centeno AC Quiroga-Centeno CA Guerrero-Macias S Navas-Quintero O Gomez-Ochoa SA . Systematic review and meta-analysis of risk factors for mesh infection following abdominal wall hernia repair surgery. Am J Surg (2022) 224(1 Pt A):239–46. 10.1016/j.amjsurg.2021.12.024 34969506

[5] Stabilini C Theodorou A Pawlak M Antoniou S Berrevoet F Bougard H EHS guidelines on the management of primary ventral and incisional hernias under emergency conditions. J Abdom Wall Surg (2026) 5:16228. 10.3389/jaws.2026.16228 41938186 PMC13044802

[6] Velmahos GC Demetriades D Mahoney E Burke P Davis K Larentzakis A The worst-case scenario: bridging repair with a biologic mesh in high-risk patients with very large abdominal wall hernias-a prospective multicenter study. Surgery (2021) 169(2):318–24. 10.1016/j.surg.2020.08.036 33066982

[7] Primus FE Harris HW . A critical review of biologic mesh use in ventral hernia repairs under contaminated conditions. Hernia (2013) 17(1):21–30. 10.1007/s10029-012-1037-8 23296600 PMC3601571

[8] Rosen MJ Krpata DM Petro CC Carbonell A Warren J Poulose BK Biologic vs synthetic mesh for single-stage repair of contaminated ventral hernias: a randomized clinical trial. JAMA Surg (2022) 157(4):293–301. 10.1001/jamasurg.2021.6902 35044431 PMC8771431

[9] Kockerling F Alam NN Antoniou SA Daniels IR Famiglietti F Fortelny RH What is the evidence for the use of biologic or biosynthetic meshes in abdominal wall reconstruction? Hernia (2018) 22(2):249–69. 10.1007/s10029-018-1735-y 29388080 PMC5978919

[10] Bueno-Lledo J Porrero-Guerrero B Ferreira F Pena-Soria MJ Sanz-Sanchez M Mansilla-Rosello A Long-term results with biosynthetic absorbable P4HB mesh in ventral abdominal wall repair: a multicentre analysis. Hernia (2024) 28(4):1103–12. 10.1007/s10029-024-02981-6 38478185

[11] Charleux-Muller D Hurel R Fabacher T Brigand C Rohr S Manfredelli S Slowly absorbable mesh in contaminated incisional hernia repair: results of a French multicenter study. Hernia (2021) 25(4):1051–9. 10.1007/s10029-020-02366-5 33492554

[12] van Rooijen MM Jairam AP Tollens T Jorgensen LN de Vries Reilingh TS Piessen G Outcomes of a new slowly resorbable biosynthetic mesh (phasix) in potentially contaminated incisional hernias: a prospective, multi-center, single-arm trial. Int J Surg (2020) 83:31–6. 10.1016/j.ijsu.2020.08.053 32931978

[13] Dempsey SG Miller CH Hill RC Hansen KC May BCH . Functional insights from the proteomic inventory of ovine forestomach matrix. J Proteome Res (2019) 18(4):1657–68. 10.1021/acs.jproteome.8b00908 30879303

[14] DeNoto G 3rd Ceppa EP Pacella SJ Sawyer M Slayden G Takata M 24-Month results of the BRAVO study: a prospective, multi-center study evaluating the clinical outcomes of a ventral hernia cohort treated with oviTex(R) 1S permanent reinforced tissue matrix. Ann Med Surg (Lond) (2022) 83:104745. 10.1016/j.amsu.2022.104745 36389188 PMC9661641

[15] Wilson P Nour HM Sajjanshetty M . BHSeP8 single unit experience of using OviTex reinforced tissue matrix in open and laparoscopic abdominal wall reconstruction (AWR). Br J Surg (2025) 112(Suppl. ment_3):znae323.012. 10.1093/bjs/znae323.012

[16] Timmer AS Claessen JJM Brouwer de Koning IM Haenen SM Belt EJT Bastiaansen A Clinical outcomes of open abdominal wall reconstruction with the use of a polypropylene reinforced tissue matrix: a multicenter retrospective study. Hernia (2022) 26(5):1241–50. 10.1007/s10029-022-02604-y 35441284 PMC9525385

[17] Ghazi B Deigni O Yezhelyev M Losken A . Current options in the management of complex abdominal wall defects. Ann Plast Surg (2011) 66(5):488–92. 10.1097/SAP.0b013e31820d18db 21372667

[18] Patel NG Ratanshi I Buchel EW . The best of abdominal wall reconstruction. Plast Reconstr Surg (2018) 141(1):113e–36e. 10.1097/PRS.0000000000003976 29280882

